# Estratégias para enfrentar a hesitação em vacinação no Brasil: síntese de evidências

**DOI:** 10.1590/S2237-96222026v35e20250466.pt

**Published:** 2026-10-12

**Authors:** Ana Paula da Costa Marques, Vanessa Terezinha Gubert, Adriano da Costa Dias, Antonio Luiz Dal Bello Gasparoto, Liara Ferreira dos Santos, Emmanuela Maria de Freitas Lopes, Karina Morales Lopes, Thayssa Maluff de Mello, Ana Cristina Atz dos Santos, Jadher Percio, Paulo Henrique Santos Andrade, Petra Santos Castro Rangel, Roberta Silva, Aurelina Aguiar Lima, Fernando Antônio Ribeiro Gusmão-Filho, Tereza Setsuko Toma, Antonio Jose Grande, Sandra Maria do Valle Leone de Oliveira

**Affiliations:** 1 Universidade Federal de Mato Grosso do Sul, Instituto de Biociências, Campo Grande, MS, Brasil; 2 Escola Nacional de Administração Pública, Diretoria de Altos Estudos, Brasília, DF, Brasil; 3 Universidade Federal de Mato Grosso do Sul, Campo Grande, MS, Brasil; 4 Fundação Oswaldo Cruz, Campo Grande, MS, Brasil; 5 Ministério da Saúde, Brasília, DF, Brasil; 6 Universidade de Brasília, Faculdade de Ciências da Saúde, Brasília, DF, Brasil; 7 Universidade de Pernambuco, Faculdade de Ciências Médicas, Recife, PE, Brasil; 8 Instituto de Saúde, Núcleo de Evidências, São Paulo, SP, Brasil; 9 Universidade Estadual de Mato Grosso do Sul, Campo Grande, MS, Brasil

**Keywords:** Hesitação Vacinal, Imunização, Estratégias de Saúde, Política Informada por Evidências, Informação de Saúde ao Consumidor, Vacilación a la Vacunación, Inmunización, Estrategias de Salud, Política informada por la Evidencia, Información sanitaria al consumidor

## Abstract

**Objetivo:**

Identificar as estratégias para enfrentar a hesitação vacinal.

**Métodos:**

Busca realizada nas bases de dados Medline, Embase, MedNar, Cochrane Library, Biblioteca Virtual em Saúde, Epistemonikos, Health Systems Evidence e CINAHL (EBSCO) entre junho de 2024 e fevereiro de 2025. Os estudos selecionados foram importados para o aplicativo Zotero e, em seguida, para o aplicativo Eppi-Reviewer. Foram incluídas revisões sistemáticas, sem restrição de período de publicação ou idioma, que abordassem estratégias de enfrentamento da hesitação vacinal em cenário urbano brasileiro e incluíssem adultos maiores de 18 anos e gestantes. A síntese dos resultados foi feita de forma narrativa. A qualidade metodológica foi avaliada utilizando a ferramenta A MeaSurement Tool to Assess systematic Reviews 2 (AMSTAR 2) e o framework PROGnosis RESearch Strategy (PROGRESS) para analisar a equidade nas estratégias de vacinação.

**Resultados:**

Foram identificados 1.376 artigos, sendo incluídas 48 revisões sistemáticas. As evidências indicaram que a estratégia para enfrentar a hesitação vacinal é a combinação de cinco ações: fortalecimento da comunicação para diversos grupos populacionais; implementação de programas de educação permanente para profissionais de saúde; ampliação do acesso da população às vacinas; aprimoramento do enfrentamento da desinformação sobre vacinas; e integração de estratégias de vacinação para gestantes.

**Conclusão:**

O enfrentamento efetivo da hesitação vacinal demanda estratégias combinadas e aponta para a necessidade de investimentos em infraestrutura para ampliar o acesso às vacinas, os processos de educação permanente para profissionais de saúde, a comunicação com a população e a inovação em tipos de comunicação e articulação intersetorial.

## Resume

**Objetivo:**

Identificar las estrategias para abordar la hesitación vacunal.

**Métodos:**

Búsqueda realizada en las bases de datos Medline, Embase, MedNar, Cochrane Library, Biblioteca Virtual en Salud, Epistemonikos, Health Systems Evidence y CINAHL (EBSCO) entre junio de 2024 y febrero de 2025. Los estudios seleccionados fueron importados al software Zotero y, posteriormente, a EPPI-Reviewer. Se incluyeron revisiones sistemáticas, sin restricción de período de publicación ni de idioma, que abordaran estrategias para enfrentar la hesitación vacunal en el contexto urbano brasileño e incluyeran a adultos mayores de 18 años y a gestantes. La síntesis de los resultados se realizó de forma narrativa. La calidad metodológica se evaluó mediante la herramienta A MeaSurement Tool to Assess Systematic Reviews 2 (AMSTAR 2) y el marco PROGnosis RESearch Strategy (PROGRESS) para analizar la equidad en las estrategias de vacunación.

**Resultados:**

Se identificaron 1.376 artículos, de los cuales se incluyeron 48 revisiones sistemáticas. Las evidencias indicaron que la estrategia para abordar la hesitación vacunal consiste en la combinación de cinco acciones: fortalecimiento de la comunicación con diversos grupos poblacionales; implementación de programas de educación permanente para profesionales de la salud; ampliación del acceso de la población a las vacunas; mejora del abordaje de la desinformación sobre vacunas; e integración de estrategias de vacunación para gestantes.

**Conclusión:**

El abordaje efectivo de la hesitación vacunal requiere estrategias combinadas y señala la necesidad de inversiones en infraestructura para ampliar el acceso a las vacunas, en procesos de educación permanente para profesionales de la salud, en la comunicación con la población y en la innovación en los medios de comunicación y en la articulación intersectorial.

## Introdução

A vacinação no Brasil apresenta avanços significativos, com destaque para a criação do Programa Nacional de Imunizações em 1973, a instituição do Sistema Único de Saúde (SUS) em 1988 e a integração do Programa Nacional de Imunizações à Secretaria de Vigilância em Saúde em 2003, o que fortaleceu sua estrutura e capacidade de gestão. Apesar desses esforços contínuos, a hesitação vacinal tem se mostrado um obstáculo. A pandemia de covid-19 exacerbou esse problema e evidenciou a desconfiança nas autoridades de saúde, as disparidades socioeconômicas e os fatores culturais, políticos e religiosos como barreiras à aceitação das vacinas [1].

A hesitação vacinal é um fenômeno complexo, caracterizado pela relutância ou recusa em se vacinar, mesmo com a disponibilidade dos serviços [2]. Além dos fatores já citados, destaca-se a disseminação de informações falsas, especialmente nas redes sociais, intensificada durante a pandemia [3].

Diante desse cenário, é imperativo que gestores públicos adotem abordagens flexíveis e contextualizadas para garantir o alcance e a efetividade da vacinação [4]. As sínteses de evidências apoiam esse processo ao subsidiar os gestores na identificação de alternativas alinhadas às oportunidades institucionais e adaptáveis às especificidades locais [5]. Trata-se de um processo dinâmico, que articula conhecimento técnico e capacidade de resposta às demandas sociais e estruturais do sistema de saúde [6]. 

Realizam-se diálogos deliberativos no processo de tradução de evidências em ações públicas. Esses diálogos, também chamados diálogos de políticas, são processos informados por síntese de evidências, em que atores interessados validam opções para enfrentar problemas públicos reais, considerando aceitabilidade, recursos disponíveis e contexto de implementação [7]. 

Esta síntese de evidências foi elaborada em resposta à chamada pública em Evidências em Saúde, com financiamento do Departamento de Ciência e Tecnologia do Ministério da Saúde e do Conselho Nacional de Desenvolvimento Científico e Tecnológico. O objetivo foi identificar ações/opções para enfrentar a hesitação vacinal, fornecendo subsídios para o desenvolvimento de políticas públicas. 

## Métodos

### Tipo de estudo e pergunta de pesquisa

Esta síntese de evidências seguiu a “Diretriz metodológica: síntese de evidências para políticas”, do Ministério da Saúde, e está apresentada conforme o Checklist para Relato de Sínteses de Evidências para Políticas [8]. A pergunta de pesquisa foi: Quais são as estratégias para lidar com a hesitação vacinal na população adulta, na área urbana no contexto brasileiro?

Considerando que o método das revisões sistemáticas continua sendo o padrão-ouro, sua metodologia foi adaptada e utilizada na elaboração de síntese de evidências, com o uso do acrônimo PCC, uma vez que o objetivo do estudo foi identificar opções, e não comparar intervenções. 

A estratégia de busca foi estruturada conforme a seguir.

- P (população, condição): adultos de 18 a 60 anos, idosos acima de 60 anos e gestantes, incluindo aquelas com menos de 18 anos.
- C (conceito): estratégias implementadas para promover a aceitação, a adesão ao calendário e as campanhas de vacinação.
- C (contexto): o cenário urbano brasileiro, incluindo fatores sociais, culturais, econômicos e políticos.

### Critérios de elegibilidade

Foram incluídas todas as revisões sistemáticas, sem restrição de período de publicação ou idioma, que abordassem estratégias de enfrentamento da hesitação vacinal em cenário urbano e incluíssem adultos maiores de 18 anos e gestantes. Revisões de diferentes países foram elegíveis, priorizando aplicabilidade ao contexto brasileiro.

Estudos teóricos, revisões narrativas, dissertações, teses, bem como artigos baseados em texto ou opinião, referentes ao contexto rural brasileiro, à hesitação vacinal internacional ou a menores de 18 anos foram excluídos.

Estudos primários foram utilizados apenas para complementar a análise de lacunas de equidade das ações de enfrentamento à hesitação vacinal e não compuseram a síntese principal das revisões.

### Fontes de informação

As buscas foram realizadas entre junho de 2024 e fevereiro de 2025. As fontes de informação consultadas foram Medline via PubMed, Embase via Elsevier, MedNar, Cochrane Library, Biblioteca Virtual em Saúde, Epistemonikos, Health Systems Evidence e Cumulative Index to Nursing and Allied Health Literature - CINAHL (EBSCO). 

### Estratégia de busca

Foi realizado um piloto de busca nas bases PubMed e Embase, e a estratégia de busca foi elaborada por AJG e SMVLO considerando as recomendações Peer Review of Electronic Search Strategies (PRESS). A estratégia de busca foi realizada considerando a combinação dos termos “Hesitação Vacinal”, “Recusa Vacinal” e “Aceitação Vacinal”, indexados ou não no Medical Subject Headings (MeSH). 

Foram utilizados os seguintes descritores: “*vaccine hesitancy*”, “*vaccination hesitancy*”, “hesitação vacinal”, “*vaccine refusal*”, “recusa vacinal”, “*vaccine acceptance*” e “aceitação vacinal”. Não foram utilizados filtros ou limites na busca. A estratégia de busca está apresentada na Tabela Suplementar 1. 

### Processo de seleção e coleta de dados

Os artigos identificados pela estratégia de busca foram importados para o aplicativo Zotero e, em seguida, para o aplicativo Eppi-Reviewer. Nesse aplicativo, foram removidas as citações duplicadas e foi realizado todo o processo de triagem e seleção dos artigos por quatro revisores de forma independente. A lista com os estudos excluídos e os motivos para a exclusão está apresentada na Tabela Suplementar 2.

Todas as etapas de leitura de títulos e resumos, leitura de textos na íntegra e extração de dados foram realizadas por duas duplas de pesquisadores (APCM e EMFL, ACD e KML). As discordâncias foram analisadas por um terceiro pesquisador (AJG).

A extração de dados foi realizada manualmente, em planilha desenvolvida pelos pesquisadores e validada pelos atores demandantes da pesquisa, com base no Apêndice E - Síntese dos Estudos por Opção da “Diretriz metodológica: síntese de evidências para políticas” [8]. A planilha do estudo continha os seguintes campos: autor/ano; opção; objetivo do estudo; características dos estudos; principais achados - contexto da revisão, resultados primários e secundários, conclusão, benefícios e danos potenciais; período do estudo; e avaliação da qualidade metodológica. 

A partir dos dados da planilha Excel, foram identificadas as opções de enfrentamento da hesitação vacinal na população adulta que vive na área urbana do Brasil, as quais foram validadas pelos atores demandantes em um diálogo de políticas. 

### Avaliação do risco de viés dos estudos e equidade em estratégias de vacinação

A avaliação da qualidade metodológica e do risco de viés foi realizada por AJG e ACD por meio da ferramenta A MeaSurement Tool to Assess systematic Reviews 2 (AMSTAR 2) [9], composta por 16 itens. Cada item foi classificado como “sim”, “não” ou “parcialmente sim”, conforme o grau de conformidade com os critérios metodológicos. A resposta “sim” indicou atendimento pleno ao item; “não” se referiu à impossibilidade de avaliação por ausência de informações; e “parcialmente sim” apontou para uma adesão parcial ao critério. Os escores foram posteriormente agrupados e categorizados em níveis de confiança metodológica: alto (0-1 item não crítico), moderado (>1 item não critico), baixo (1 item crítico) ou criticamente baixo (>1 item crítico).

Estudos primários foram incluídos apenas para complementar a análise da equidade das ações de enfrentamento à hesitação vacinal e não compuseram a síntese principal das revisões. O framework PROGnosis RESearch Strategy (PROGRESS) [10] foi utilizado para ajudar a identificar barreiras e oportunidades para promover equidade no acesso às vacinas. Foram avaliadas as dimensões local de residência, raça, ocupação, gênero/sexo, educação, condição socioeconômica e capital social. Esta análise foi realizada de forma independente por AJG e ACD.

### Método de síntese

Os dados foram sumarizados por meio de síntese narrativa, com o agrupamento das estratégias identificadas em categorias temáticas previamente definidas:

- opção 1 - fortalecer a comunicação para os diversos grupos populacionais;
- opção 2 - implementar programas de educação permanente para os profissionais de saúde;
- opção 3 - ampliar o acesso da população às vacinas;
- opção 4 - aprimorar o enfrentamento da desinformação sobre vacinas; e
- opção 5 - integrar estratégias de vacinação para gestantes.

Essa abordagem permitiu organizar os achados de forma descritiva, destacando os tipos de intervenção, os públicos-alvo e os contextos de aplicação.

A sumarização considerou a frequência com que cada estratégia foi relatada nos estudos incluídos, bem como os efeitos observados sobre a adesão vacinal. As evidências foram apresentadas de forma qualitativa, sem meta-análise, devido à heterogeneidade dos desenhos metodológicos, populações estudadas e indicadores de resultado. Além disso, foram destacados os fatores contextuais que influenciam a efetividade das estratégias, como aspectos culturais, sociais e econômicos.

## Resultados

A busca inicial identificou 1.376 artigos, sendo triadas 1.055 revisões após a retirada de duplicatas. Durante a leitura completa de 55 artigos, 7 foram excluídos e estão listados na Tabela Suplementar 2. No total, foram incluídas 48 revisões sistemáticas (Figura 1). 

Publicadas entre 2012 e 2024, as revisões foram conduzidas em países de alta renda, com evidências também de países de média e baixa renda, incluindo o Brasil. As vacinas abordadas incluíram covid-19, influenza, papilomavírus humano (HPV), hepatite B, coqueluche e pneumococo, com foco em populações e contextos diversos (Tabela 1). 

Cada revisão descreveu de uma a quatro ações de enfrentamento à hesitação vacinal, sendo a combinação de estratégias a mais efetiva. As cinco principais ações identificadas foram: fortalecer a comunicação com diferentes grupos; implementar programas de educação permanente para os profissionais de saúde; ampliar o acesso às vacinas; combater a desinformação; e integrar estratégias para gestantes. Essas ações foram validadas com os atores demandantes em diálogo de políticas e ao final da elaboração da síntese de evidências para o Programa Nacional de Imunizações.

Diversas estratégias têm sido adotadas para fortalecer a comunicação, promover a adesão à vacinação e mitigar a hesitação vacinal [42]. O uso de teorias de mudança de comportamento, especialmente o modelo de crenças em saúde, tem se destacado. Entre mídias sociais, predominam postagens educativas, seguidas por grupos de diálogos, sites interativos e lembretes pessoais [39]. A comunicação honesta e transparente, aliada à informação sobre os benefícios e os possíveis eventos adversos, fortalece a credibilidade das campanhas de vacinação [34]. O contato direto pode ser eficaz para públicos com resistência à vacinação [19]. 

**Figura 1:**
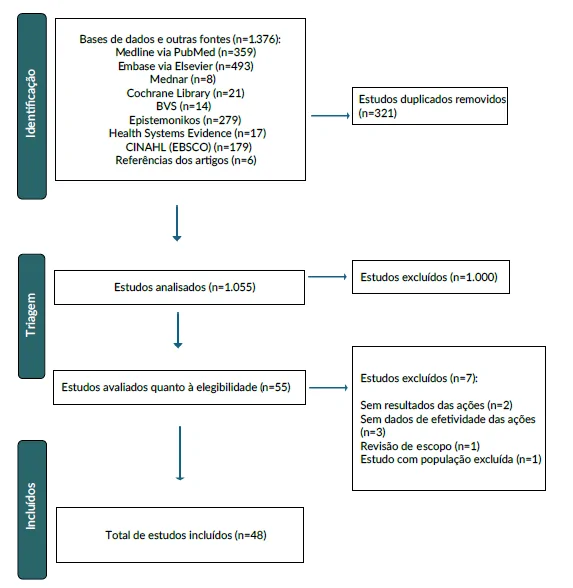
Processo de seleção e inclusão dos estudos

A comunicação sensível às nuances culturais e pessoais é mais eficaz do que estratégias baseadas exclusivamente em conhecimentos epidemiológicos e médicos [42]. O envolvimento de líderes locais e religiosos fortalece a confiança e a adesão, especialmente entre minorias étnicas e migrantes. Intervenções com técnicas de mudança comportamental, aliadas a mensagens adaptadas e apoio de organizações comunitárias, mostraram-se eficazes para alcançar esses grupos [34]. 

Estratégias multifacetadas, como cartazes, folhetos, comunicação interna e oferta ativa de vacinas no local de distribuição desses materiais, aumentaram significativamente a taxa de vacinação contra a gripe. Vídeos, realidade virtual e aplicativos móveis tornam a comunicação mais dinâmica e atrativa, especialmente entre jovens, e complementam as abordagens tradicionais, potencializando o engajamento e a adesão [19]. Destacam-se atividades educativas para usuário, rastreamento nas carteiras de vacinação por agentes comunitários de saúde e intervenções simultâneas direcionadas a unidades de saúde, aos profissionais (educação de pais, avisos, auditoria e feedback) e a ambientes familiares (telefonemas) [59].

Entre as mídias sociais, postagens educacionais, grupos que interagem com diálogos, sites interativos e lembretes pessoais são os mais utilizados. As estratégias foram embasadas na teoria do nudge (ou empurrãozinho) [57]. Essa teoria propõe influenciar as escolhas de forma sutil, sem restringir opções nem impor obrigações, introduzindo pequenas alterações no ambiente ou no contexto em que as decisões são tomadas. 

**Tabela 1:** Características dos estudos incluídos (n=48)

Referência	Opção	Principais achados	Qualidade metodológica
[11]	3 e 4	Recomenda-se construir confiança, reduzir barreiras físicas, melhorar a comunicação e a transparência sobre o desenvolvimento das vacinas com o apoio de profissionais de saúde, líderes religiosos e comunitários, para ampliar o acesso e a adesão entre minorias étnicas e migrantes.	Alta
[12]	1	Para aumentar a aceitação da vacina, deve-se considerar contextos socioculturais, históricos e políticos, priorizando comunicação, engajamento comunitário e recursos culturalmente inclusivos, com estratégias personalizadas que reduzem a hesitação e aumentam a adesão à vacinação.	Moderada
[13]	1	Estratégias que utilizam tecnologias digitais têm impacto modesto e positivo na aceitação da vacina e na conclusão da série de vacinação em comparação com intervenções não digitais.	Alta
[14]	1	O acesso ao prontuário eletrônico aumenta de forma moderada a aceitação da vacinação, e sua combinação com recursos de comunicação digital potencializa esse efeito, superando o impacto do acesso isolado ao prontuário eletrônico.	Moderada
[15]	3	Comunicações personalizadas e lembretes por mensagem aumentam a adesão à vacinação, enquanto comunicar incertezas sobre a vacina não reduz a intenção de se vacinar, mas pode gerar impacto negativo.	Alta
[16]	2 e 4	Políticas futuras devem responder às preocupações da população, promover compreensão sobre as vacinas, neutralizar a desinformação e ampliar a aceitação, com treinamento baseado em evidências sobre vacinas e estratégias de vacinação para profissionais de saúde.	Alta
[17]	5	Várias estratégias foram consideradas eficazes: lembretes sobre vacinação em registros de saúde pré-natal, parteiras que fornecem vacinação e educação e fornecimento de informações para a equipe de saúde e usuários.	Moderada
[18]	5	Intervenções educativas, como panfletos, websites e sessões individuais breves, aumentam a cobertura vacinal contra a influenza em gestantes, com maior aceitação da vacina contra a gripe	Moderada
[19]	1 e 2	Intervenções de comunicação promocional mais eficazes são aquelas que são personalizadas, utilizam múltiplos canais, envolvem profissionais de saúde, são interativas e abordam as preocupações do público.	Moderada
[20]	1	Barreiras como idioma, acesso precário a cuidados médicos e desconfiança no sistema médico são modificáveis ​​com maior comprometimento de recursos. Promover políticas que garantirão financiamento federal para serviços de saúde e adoção de diretrizes do Comitê Consultivo sobre Práticas de Imunização, melhorará as taxas de vacinação para refugiados, imigrantes e migrantes.	Moderada
[21]	3	Entre profissionais de saúde, palestras, materiais informativos, unidades móveis, horários estendidos e engajamento de líderes são estratégias eficazes. A combinação com incentivos motivacionais fortalece a adesão, sendo essencial em contextos em que a vacinação obrigatória gera controvérsia.	Moderada
[22]	1 e 2	Intervenções eficazes para aumentar a adesão à vacinação demandam abordagens personalizadas e culturalmente sensíveis. A não eficiência de modelos universais ressalta a necessidade de ações adaptadas ao público, considerando seus costumes e ideologias.	Alta
[23]	1	Os estudos revisados ​​incluíram intervenções com conteúdo e abordagens diversos que foram implementadas em diferentes cenários e direcionadas a várias populações. Poucas intervenções foram direcionadas diretamente a indivíduos em hesitação vacinal.	Moderada
[24]	1, 2 e 5	Intervenções multicomponentes, adaptadas às necessidades de cada minoria étnica, mostram eficácia ao aumentar a aceitação da vacina, com destaque para a conscientização e o envolvimento de organizações comunitárias.	Alta
[25]	2	Intervenções multidimensionais baseadas na comunidade, na família e na assistência médica, aliadas a estratégias de educação, melhoram efetivamente a cobertura de vacinação. A implementação sistemática pode ser relevante para atingir comunidades subvacinadas.	Alta
[26]	1	E-mails aumentam a aceitação da vacina em comparação com a ausência de intervenção. Métodos combinados e personalizados de lembrete mostram-se mais eficazes. A personalização e a integração de diferentes estratégias são cruciais para otimizar os resultados.	Alta
[27]	2 e 3	Iniciativas de educação por pares têm potencial para melhorar a aceitação da vacina, sendo mais eficazes quando combinam pares e especialistas em saúde, com ações específicas e de longo prazo.	Alta
[28]	2	As estratégias variaram amplamente em modo de entrega e técnicas de mudança de comportamento. Após as intervenções, as taxas de vacinação de influenza, pneumocócica e herpes-zóster aumentaram em 16,6% em um período de 12 a 16 meses.	Moderada
[29]	2	A imunidade coletiva representa um benefício significativo da vacinação em nível populacional. Comunicar esse benefício de forma otimizada é crucial, pois há indícios de que uma comunicação eficaz sobre a imunidade coletiva pode aumentar as intenções de vacinação.	Alta
[30]	4	As mídias sociais têm potencial para disseminar informações e estimular a vacinação, mas sua eficácia exige monitoramento ativo e responsável das autoridades e deve ser combinada com outras estratégias, dada a complexidade da aceitação vacinal.	Moderada
[31]	3	Intervenções comportamentais, financeiras ou não, têm efeito positivo modesto na aceitação da vacina, com impacto semelhante entre ações únicas e combinadas O momento da implementação não altera os resultados, exceto em intervenções coletivas, que podem perder eficácia com a evolução da covid-19.	Alta
[32]	2	Quando farmacêuticos participaram do processo de imunização, independentemente de sua função ou do tipo de vacina administrada, a cobertura vacinal foi maior.	Alta
[33]	3	Intervenções multicomponentes e baseadas em diálogo foram mais eficazes, mas, dadas a complexidade da hesitação vacinal e as evidências limitadas, as estratégias identificadas devem ser adaptadas à população-alvo, às suas razões para hesitação e ao contexto específico.	Alta
[34]	1, 2, 3 e 5	As estratégias mais eficazes incluem informações claras, lembretes personalizados, melhoria no acesso à vacinação e incentivo dos profissionais de saúde. No Reino Unido, focar a relevância da informação e a melhoria do acesso se mostrou particularmente eficaz.	Alta
[35]	3	As barreiras à vacinação variam por doença infecciosa e população; os principais motivadores são a proteção pessoal, familiar e da comunidade. Para a influenza, estratégias mais eficazes focam conveniência e confiança, abordando questões como custo e educação.	Moderada
[36]	2, 3 e 5	A aceitação da vacinação em gestantes foi facilitada pela recomendação de profissionais de saúde, conhecimento, confiança na vacina, múltiplas consultas de pré-natal, e estratégias como financiamento, lembretes, integração de serviços, engajamento comunitário, capacitação e educação.	Moderada
[37]	5	A adesão à vacinação pós-parto é fortalecida por: recomendação de profissionais de saúde, intervenções adequadas, aplicação em maternidades ou comunidades e educação focada no reforço positivo.	Moderada
[38]	1, 2 e 5	Informar riscos e segurança da vacina, desmistificar equívocos, usar lembretes e campanhas mistas, além de mensagens curtas e claras adaptadas ao público, aumentam a aceitação e fortalecem crenças positivas sobre vacinação.	Baixa
[39]	1 e 2	Mensagens educacionais aumentam o conhecimento, mas intervenções dialógicas nas redes sociais, fundamentadas em teoria da mudança de comportamento e adaptadas ao público, mostram maior potencial para promover a vacinação e reduzir a hesitação.	Moderada
[40]	4	As intervenções em mídias sociais são uma área de estudo emergente e promissora para aumentar a aceitação da vacina, nas quais o enquadramento da mensagem, a credibilidade do emissor e a estrutura da rede social são fatores decisivos, tornando essencial otimizar seu uso.	Moderada
[41]	2	Aplicativos, jogos e simulações são promissoras intervenções para aprimorar o conhecimento e a confiança de profissionais de saúde e estudantes na vacinação, com melhores resultados quando combinadas com ações direcionadas também aos usuários.	Alta
[42]	1,2 e 3	Comunicações diversas e combinadas, incluindo métodos de massa e abordagens personalizadas com melhores serviços, mostram potencial para aumentar a aceitação da vacina, especialmente contra a gripe em idosos.	Moderada
[43]	1 e 3	Participantes tinham mais probabilidade de se vacinar quando incentivados por dinheiro. Os efeitos de outras loterias e mensagens persuasivas tiveram pouco efeito ou não foram significativos.	Moderada
[44]	1 e 2	Estratégias eficazes de promoção de vacinação incluem comunicação (webinars, mídias sociais e materiais educativos), parcerias entre estados, universidades e líderes comunitários, além de intervenções de distribuição como prefeituras virtuais, linhas diretas confidenciais, materiais educativos e oferta de vacinação durante consultas médicas de rotina.	Moderada
[45]	1 e 2	Reforçar o papel dos profissionais de saúde na promoção da vacinação é crucial. Estratégias como ordens permanentes, lembretes e esforços de recall são eficazes. Farmácias comunitárias, especialmente em áreas remotas, têm potencial para melhorar a cobertura vacinal.	Moderada
[46]	1 e 2	Intervenções baseadas em jogos geralmente aumentam o conhecimento sobre vacinação. Contudo, o impacto na adesão à vacinação em si é limitado. A personalização de avatares e a colaboração em jogos se mostraram eficazes para engajar os jogadores.	Moderada
[47]	1	Vídeos, realidade virtual e aplicativos com redes sociais ou gamificação mostraram-se mais eficazes que materiais impressos para aumentar o conhecimento, a confiança e o engajamento na vacinação.	Alta
[48]	1	*Chatbots* de vacinas têm sido usados para fornecer informações factuais, agendar vacinações, enviar lembretes e enfrentar a desinformação, assim os *chatbots* mostram potencial para influenciar positivamente as atitudes relacionadas às vacinas.	Moderada
[49]	1	As estratégias multicomponentes foram mais eficientes, principalmente quando direcionadas a múltiplos determinantes da vacinação.	Baixa
[50]	5	Recomendações claras e personalizadas para usuários, educação sobre segurança e estímulo à oferta de vacinas para provedores e incentivos financeiros, registros eletrônicos de saúde e disponibilidade da vacina no nível político são eficazes.	Alta
[51]	1	Intervenções de decisão compartilhada aumentaram significativamente a aceitação das vacinas em relação ao grupo controle.	Moderada
[52]	1, 2 e 3	Intervenções comunitárias, incentivos financeiros e programas de alfabetização em saúde baseados em tecnologia aumentaram o uso de serviços de imunização. Intervenções baseadas em mídia não demonstraram ser eficazes para reduzir a hesitação vacinal.	Alta
[53]	1 e 2	Intervenções não financeiras, como lembretes de consultas e sistemas de agendamento de *opt-out*, parecem encorajar a aceitação da vacina. Outras abordagens, como intervenções multimodais e infográficos, também mostram potencial para enfrentar a hesitação vacinal.	Alta
[54]	3	As estratégias-chave para promover a vacinação incluem compreender e colaborar com as comunidades, personalizar mensagens, abordar traumas sistêmicos e lacunas de saúde social e realizar planejamento logístico antecipado.	Alta
[55]	2	Intervenções multicomponentes foram eficazes para aumentar as taxas de vacinação, seguidas por lembretes personalizados, embora menos eficazes que ordens permanentes ou lembretes ao usuário.	Alta
[56]	1	Estratégias eficazes contra hesitação vacinal incluem: mensagens normativas, fontes com identidade compartilhada, canais interativos (como *chatbots* e vídeos), informações sobre segurança e eficácia, apelos coletivos com constrangimento e apoio de líderes políticos.	Alta
[57]	1	Intervenções baseadas na teoria do nudge aumentaram a taxa de aceitação da vacina contra a covid-19, sendo eficazes quando combinavam múltiplos componentes (normas sociais e lembrete), embora seus efeitos diminuíssem ao longo do tempo.	Alta
[58]	4	A desinformação relacionada à vacina foi abordada em cinco etapas: identificação de desinformação, regulamentação de produtores e distribuidores, corte de produção e distribuição, suporte a públicos-alvo e disseminação de informações confiáveis.	Alta

O sucesso das estratégias de comunicação depende da integração de múltiplas abordagens, considerando aspectos culturais, tecnológicos e sociais. A combinação de métodos tradicionais com o uso de tecnologia digital, aliada à adaptação cultural e linguística das mensagens, favorece o alcance de diferentes grupos e mudanças efetivas de comportamentos relacionados à saúde.

A educação permanente em saúde é uma política do SUS que deve ser utilizada como opção para enfrentar a hesitação vacinal. A formação do profissional de saúde deve desenvolver nele habilidades de comunicação para transmitir, com clareza e objetividade, as informações à população e para engajar outros profissionais na prescrição e recomendação de vacinas indicadas pelo Programa Nacional de Imunizações [59]. Narrativas pessoais combinadas com informações médicas claras e acessíveis foram efetivas para engajar diferentes públicos, desde estudantes universitários até a população em geral [39]. 

Lembretes personalizados para médicos, como uma forma de educação passiva, têm demonstrado efeitos positivos [55]. Mensagens curtas, com foco na redução de risco, no reforço dos benefícios sociais da vacinação e na abordagem de crenças e preocupações das populações-alvo, como segurança da vacina, ampliam o impacto comportamental [38].

A dificuldade de acesso aos serviços de saúde contribui para a hesitação vacinal. Melhorar a sinalização das salas de vacina e divulgar horários de funcionamento dos postos de vacinação são medidas relevantes [15]. Unidades móveis, clínicas de vacinação e horários estendidos ampliam o acesso de trabalhadores e comunidades remotas [21,34]. A avaliação de barreiras e motivadores mostra que a vacinação em locais não tradicionais, como escolas, empresas e centros comunitários, tem se mostrado eficaz [35].

A desinformação é um dos maiores obstáculos às campanhas de vacinação, tornando essencial enfrentá-la para reduzir a hesitação vacinal [25]. A atuação ativa e confiável dos órgãos oficiais nas redes sociais, com respostas transparentes, incentivo à divulgação de informações verdadeiras e checagem de fatos por profissionais de saúde, é fundamental para enfrentar a desinformação e aumentar a credibilidade nas vacinas [30,40]. Para viabilizar essas estratégias, faz-se necessário criar sistemas de vigilância, regular a produção de informação e estabelecer normas contra a circulação de conteúdos falsos [58].

A vacinação de gestantes e lactantes ainda representa um desafio de saúde pública, com taxa de cobertura de 48,4% atribuída à falta de informação, à percepção de insegurança e ao medo de eventos adversos para a mãe e o bebê. No caso da covid-19, além das razões tradicionais, gestantes descreveram desconfiança quanto à eficácia e à segurança das vacinas, influenciada pela rapidez no desenvolvimento e pela percepção de interferência das empresas farmacêuticas nas políticas públicas [60]. 

Estratégias que combinaram mensagens afetivas, como vídeos e livros, com aplicações cognitivas aumentaram o conhecimento e a intenção de vacinar, sendo vídeo o recurso mais eficaz [24]. Estratégias educacionais multimodais, de informações orais, escritas e visuais, como cartazes, podem aumentar as taxas de vacinação. 

A qualificação dos profissionais de saúde é essencial para enfrentar a hesitação vacinal. A formação deve incluir estratégias para esclarecer dúvidas das gestantes e fortalecer a confiança na vacinação, cuja recomendação médica é um determinante [37]. Toda comunicação deve ser clara, incluir benefícios para o bebê e ser adequada ao momento gestacional, considerando as necessidades e as preocupações da mulher [34]. 

Facilitar o acesso à vacinação, especialmente onde se encontram gestantes, como maternidades, favorece a adesão à vacinação [37]. Intervenções educacionais, mensagens de texto e ações combinadas têm mostrado efeito positivo na imunização materna em contextos de baixa renda [18,36]. 

A avaliação das 48 revisões sistemáticas com a ferramenta AMSTAR 2 indicou 50,0% com alta qualidade metodológica e 45,8% com qualidade moderada; 4,2% foram classificadas como de baixa qualidade. Essa distribuição confere robustez à síntese, embora exija atenção às limitações metodológicas na interpretação dos achados. A Tabela 2 apresenta a avaliação do risco de viés. 

**Tabela 2:** Avaliação do risco de viés e da qualidade metodológica (n=48)

	Dezesseis perguntas que compõem a ferramenta AMSTAR 2																
Autor, ano	1	2	3	4	5	6	7	8	9	10	11	12	13	14	15	16	Classificação
Abba-Aji, 2022 [11]	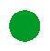	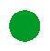	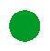	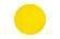	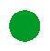	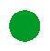	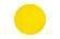	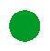	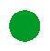	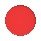	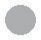	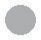	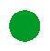	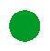	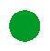	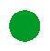	Alta
Adeagbo, 2022 [12]	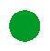	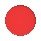	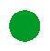	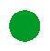	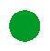	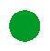	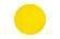	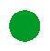	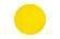	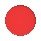	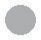	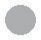	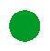	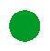	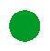	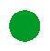	Moderada
Atkinson, 2019 [13]	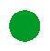	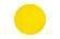	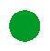	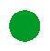	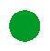	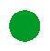	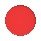	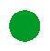	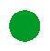	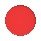	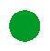	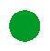	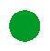	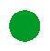	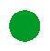	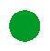	Alta
Balzarini, 2020 [14]	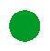	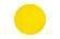	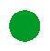	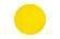	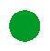	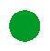	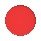	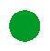	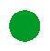	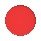	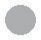	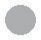	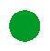	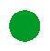	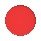	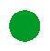	Moderada
Batteux, 2022 [15]	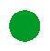	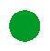	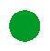	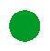	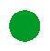	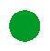	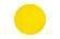	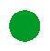	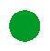	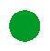	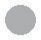	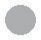	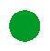	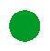	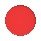	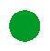	Alta
Begum, 2024 [16]	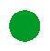	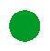	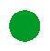	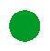	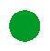	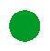	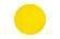	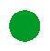	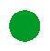	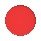	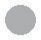	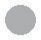	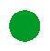	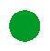	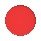	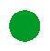	Alta
Bisset e Paterson, 2018 [17]	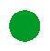	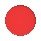	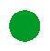	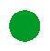	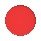	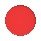	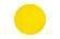	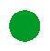	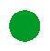	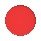	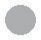	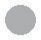	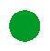	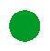	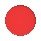	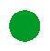	Moderada
Brillo, Tosto e Buonomo, 2023 [18]	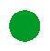	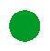	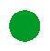	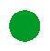	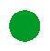	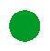	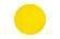	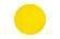	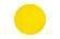	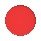	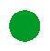	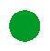	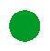	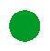	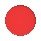	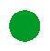	Moderada
Cairns, 2012 [19]	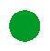	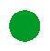	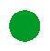	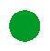	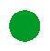	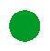	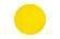	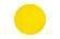	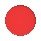	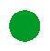	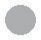	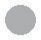	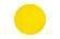	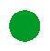	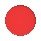	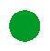	Moderada
Daniels, 2022 [20]	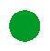	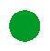	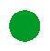	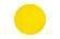	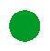	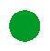	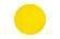	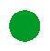	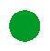	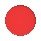	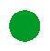	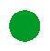	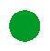	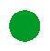	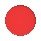	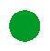	Moderada
De Koning, 2024 [21]	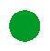	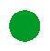	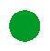	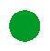	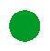	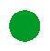	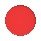	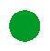	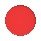	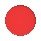	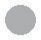	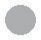	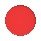	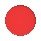	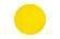	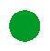	Moderada
Desjardins, 2023 [22]	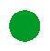	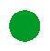	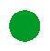	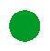	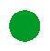	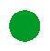	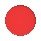	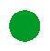	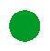	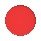	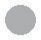	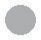	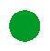	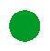	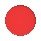	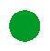	Alta
Dubé, Gagnon e MacDonald, 2015 [23]	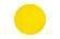	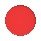	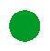	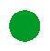	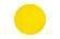	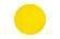	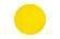	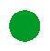	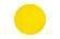	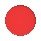	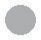	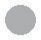	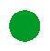	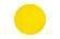	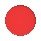	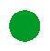	Moderada
Ekezie, 2023 [24]	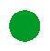	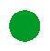	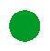	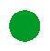	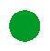	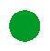	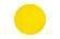	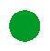	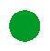	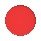	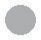	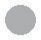	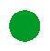	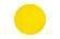	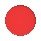	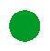	Alta
Fallucca, 2024 [25]	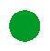	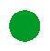	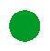	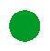	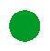	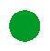	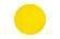	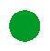	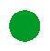	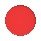	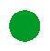	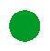	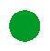	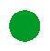	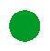	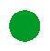	Alta
Frascella, 2020 [26]	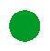	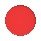	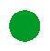	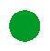	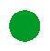	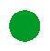	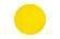	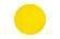	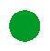	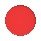	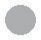	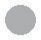	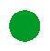	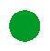	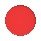	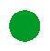	Alta
Gobbo, 2023 [27]	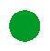	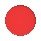	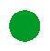	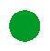	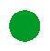	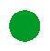	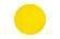	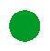	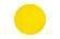	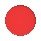	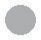	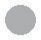	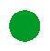	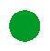	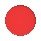	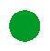	Alta
Gosselin Boucher, 2019 [28]	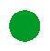	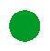	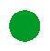	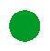	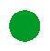	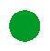	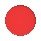	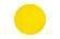	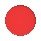	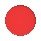	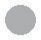	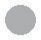	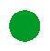	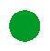	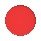	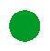	Moderada
Hakim, 2019 [29]	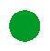	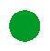	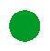	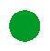	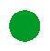	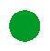	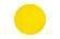	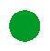	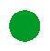	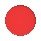	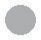	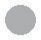	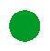	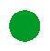	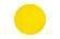	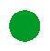	Alta
Hansen, Baiju e Gabarron, 2023 [30]	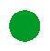	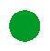	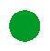	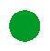	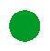	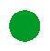	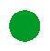	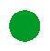	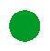	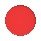	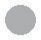	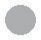	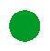	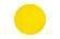	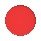	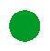	Moderada
Huang, Huang e Yu, 2023 [31]	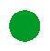	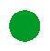	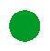	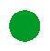	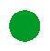	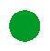	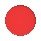	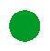	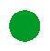	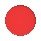	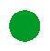	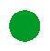	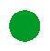	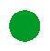	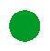	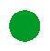	Alta
Isenor, 2016 [32]	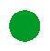	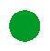	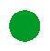	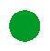	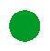	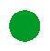	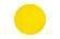	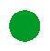	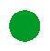	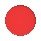	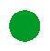	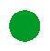	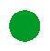	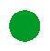	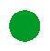	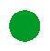	Alta
Jarrett, 2015 [33]	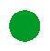	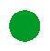	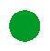	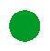	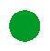	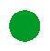	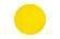	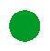	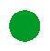	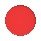	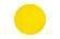	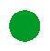	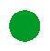	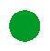	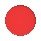	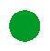	Alta
Kafadar, 2024 [34]	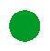	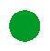	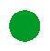	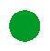	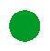	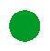	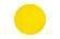	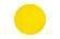	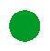	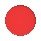	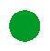	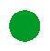	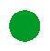	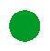	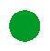	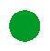	Alta
Kalunga, 2023 [35]	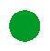	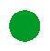	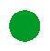	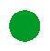	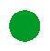	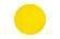	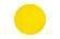	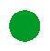	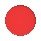	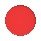	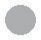	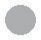	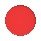	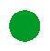	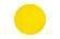	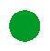	Moderada
Khan, 2024 [36]	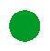	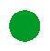	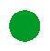	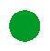	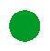	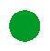	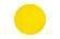	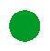	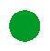	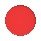	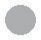	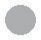	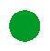	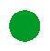	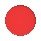	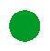	Moderada
Konstantinou, 2024 [37]	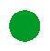	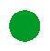	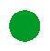	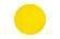	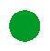	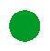	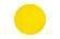	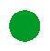	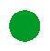	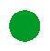	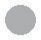	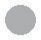	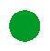	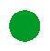	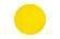	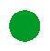	Moderada
Lawes-Wickwar, 2021 [38]	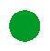	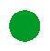	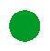	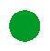	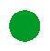	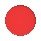	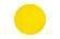	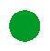	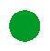	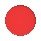	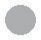	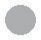	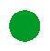	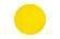	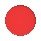	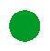	Baixa
Li, Wood e Kostkova, 2022 [39]	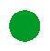	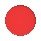	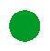	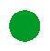	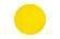	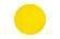	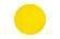	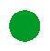	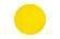	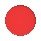	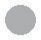	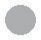	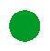	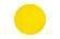	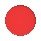	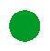	Moderada
Limaye, 2021 [40]	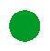	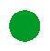	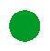	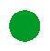	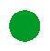	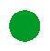	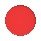	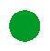	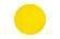	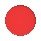	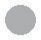	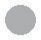	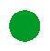	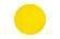	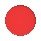	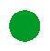	Moderada
Lo Moro, 2023 [41]	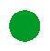	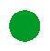	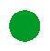	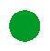	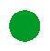	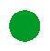	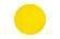	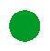	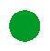	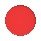	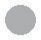	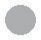	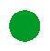	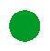	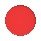	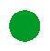	Alta
MacDonald, 2013 [42]	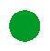	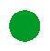	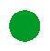	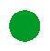	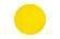	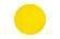	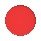	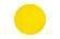	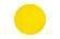	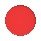	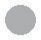	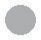	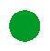	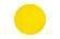	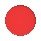	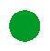	Moderada
Mardi, 2022 [43]	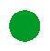	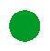	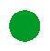	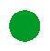	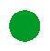	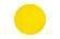	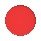	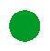	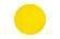	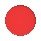	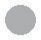	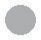	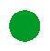	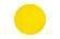	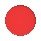	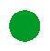	Moderada
Masterson, Anderson e Savoia, 2024 [44]	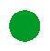	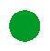	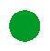	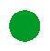	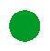	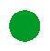	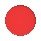	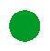	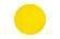	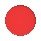	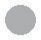	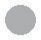	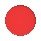	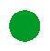	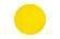	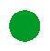	Moderada
Mohammed*,* 2021 [45]	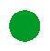	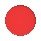	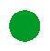	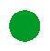	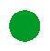	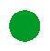	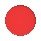	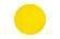	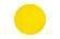	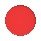	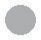	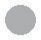	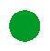	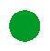	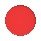	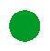	Moderada
Ou, 2024 [46]	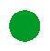	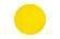	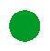	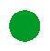	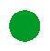	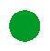	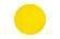	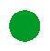	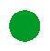	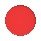	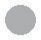	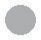	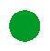	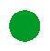	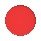	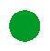	Moderada
Panickar, 2023 [47]	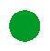	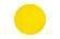	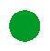	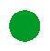	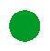	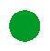	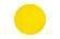	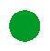	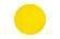	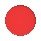	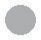	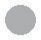	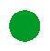	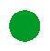	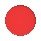	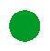	Alta
Passanante, 2023 [48]	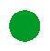	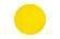	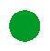	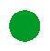	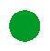	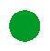	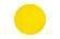	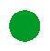	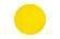	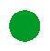	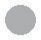	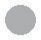	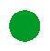	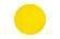	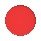	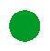	Moderada
Perroud, 2022 [49]	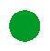	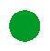	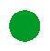	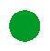	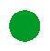	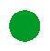	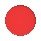	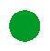	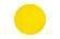	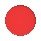	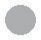	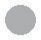	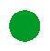	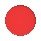	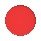	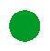	Baixa
Razai, 2023 [50]	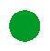	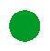	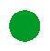	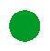	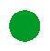	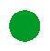	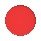	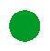	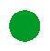	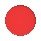	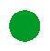	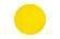	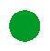	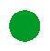	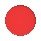	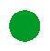	Alta
Scalia, Durand e Elwyn, 2022 [51]	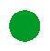	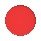	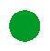	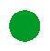	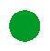	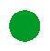	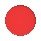	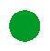	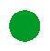	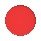	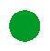	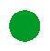	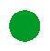	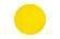	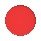	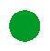	Moderada
Singh, 2022 [52]	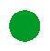	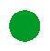	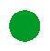	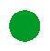	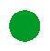	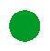	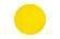	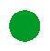	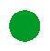	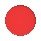	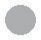	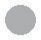	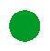	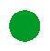	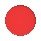	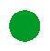	Alta
Terrell, Alami e Krewski, 2023 [53]	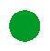	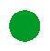	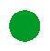	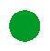	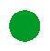	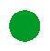	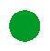	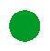	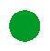	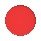	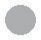	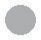	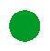	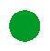	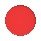	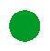	Alta
Tinessia, 2024 [54]	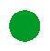	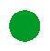	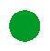	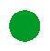	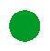	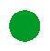	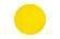	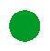	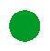	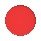	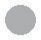	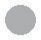	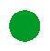	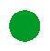	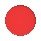	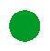	Alta
Wennekes, 2024 [55]	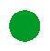	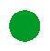	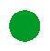	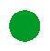	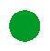	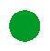	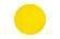	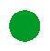	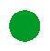	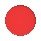	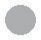	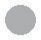	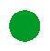	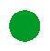	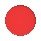	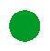	Alta
Xia e Nan, 2024 [56]	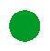	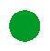	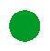	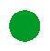	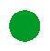	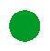	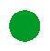	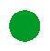	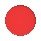	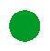	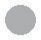	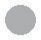	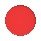	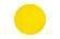	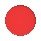	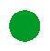	Alta
Zhang e Jin, 2024 [57]	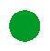	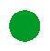	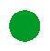	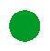	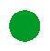	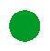	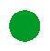	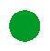	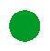	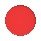	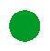	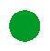	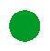	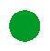	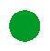	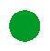	Alta
Zhao*,* 2023 [58]	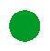	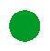	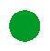	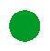	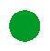	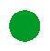	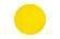	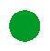	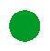	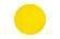	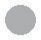	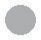	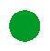	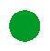	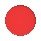	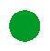	Alta

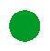
 - Sim

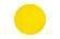
 - Parcialmente sim

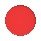
 - Não

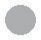
 - Não se aplica

A avaliação de políticas e programas de vacinação precisa considerar seus efeitos nas desigualdades sociais e garantir acesso a informações confiáveis e compreensíveis, independentemente de origem ou condição social. Superar barreiras históricas, culturais e tecnológicas é fundamental para alcançar a equidade, e o framework PROGRESS contribui na identificação de barreiras e oportunidades [60]. 

Regiões com baixo índice de desenvolvimento humano municipal (IDH-M) enfrentam desafios de infraestrutura e conectividade nas salas de vacina, enquanto áreas com IDH-M mais alto apresentam melhores condições logísticas. O Maranhão, apesar de apresentar um dos menores IDH da Federação, alcançou avanços na cobertura vacinal durante a pandemia da covid-19, enquanto resultado da maior cobertura da Estratégia Saúde da Família e da descentralização dos serviços de saúde [61]. Programas de imunização em comunidades remotas têm se mostrado fundamentais para promover a equidade [62].

Populações minoritárias e grupos étnicos enfrentam desafios históricos e culturais de desconfiança nas intervenções clínicas, decorrentes de exclusão ou discriminação. A comunicação deve ser adaptada aos contextos culturais e linguísticos para garantir a compreensão efetiva [16]. Estratégias com participação comunitária, uso de materiais informativos claros e baseados em evidências fortalecem a confiança e a aceitação da vacina entre gestantes [10]. Ações como busca ativa e vacinação no local de atendimento têm sido eficazes em populações vulneráveis [11].

Gestantes apresentam vulnerabilidades específicas, exigindo estratégias direcionadas devido a alterações imunológicas durante a gravidez. A qualidade do pré-natal influencia diretamente nas taxas de vacinação, com desigualdades mais acentuadas entre mulheres de baixa renda e escolaridade. A hesitação vacinal nesse grupo decorre da falta de informação confiável, das crenças de insegurança das vacinas e do medo de efeitos adversos [30]. Materiais informativos, sessões individuais e recursos digitais demonstraram ser eficazes em aumentar a adesão nesse grupo e promover a equidade no cuidado materno-infantil [18,63]. Promover maior equidade no cuidado à saúde materno-infantil exige políticas que enfrentem barreiras socioeconômicas e educacionais, promovam o acesso e fortaleçam os sistemas de saúde na proteção de gestantes e recém-nascidos [10,18,61,63].

O nível educacional mostra forte correlação com a adesão à vacinação, sendo necessário o uso de materiais acessíveis e linguagem simplificada para populações com baixa alfabetização [64]. O letramento digital em saúde emerge como ferramenta estratégica, especialmente durante pandemias [65]. A roda de conversa se mostrou eficiente para estimular a vacinação em grupos vulneráveis, como usuários e acompanhantes em sala de espera de serviços oncológicos [1]. 

A promoção da participação social é essencial para enfrentar desigualdades no acesso à saúde, favorecendo grupos vulneráveis [53]. Sob a perspectiva PROGRESS, recomenda-se fortalecer a infraestrutura em áreas de baixo IDH-M; desenvolver comunicação culturalmente sensível e adaptada; implementar programas para grupos ocupacionais vulneráveis; garantir atenção à saúde materna e à vacinação no pré-natal; promover educação em saúde e letramento digital; reduzir barreiras socioeconômicas ao acesso; e ampliar redes comunitárias e parcerias intersetoriais. O acesso equitativo à vacinação exige considerar as múltiplas barreiras enfrentadas por diferentes grupos.

## Discussão

Questões culturais, sociais, econômicas e informacionais influenciam a hesitação vacinal. Para enfrentar tal cenário, sugere-se fortalecer a comunicação com diversos grupos populacionais, implementar educação permanente para os profissionais de saúde, ampliar o acesso às vacinas, combater a desinformação sobre vacinas e integrar estratégias específicas para gestantes [40-41].

O fortalecimento da comunicação exige o envolvimento de líderes comunitários e a adaptação da linguagem aos valores locais. As mensagens devem destacar benefícios coletivos e individuais da vacinação, com uso de apelos emocionais e figuras políticas para criar identificação e confiança [42]. Campanhas de mídia, educação comunitária e expansão do acesso aos serviços de vacinação são fundamentais, assim como os esforços contínuos de sensibilização realizados pelas equipes de saúde. Tecnologias digitais, como lembretes eletrônicos, plataformas on-line, serviço de mensagens curtas (SMS), redes sociais e *chatbots*, ampliam o agendamento das vacinas e facilitam o acesso a informações claras sobre a segurança vacinal, alcançando um público amplo e diversificado.

Programas de educação permanente para profissionais de saúde colaboram para o enfrentamento da hesitação vacinal ao utilizar materiais visuais claros e fontes confiáveis, o que fortalece a confiança entre profissionais e usuários. Esses programas devem buscar aumentar a competência para intervenções adaptadas ao contexto local, com anúncio presuntivo em consultas. É essencial que os programas de educação permanente envolvam profissionais de saúde líderes e priorizem atividades educativas presenciais. Lembretes personalizados combinados com recursos que facilitem o trabalho nos serviços de saúde também são recomendados.

Para enfrentar a hesitação vacinal, é necessário ampliar o acesso às vacinas e melhorar a conveniência do serviço, como estender os horários de vacinação, enviar mensagens personalizadas, utilizar unidades móveis e vacinar em locais não tradicionais, como escolas e empresas. A vacinação oportunística, em ambientes de trabalho e com incentivos monetários, também aumenta a adesão.

Informações falsas são um dos maiores obstáculos às campanhas de vacinação. Diversas estratégias têm se mostrado eficazes no enfrentamento desse desafio, como o engajamento comunitário, a participação de líderes religiosos e comunitários, o agendamento automatizado, as comunicações personalizadas, as estratégias para evitar o efeito inverso e a adaptação da linguagem às preocupações do público, com regulamentação, monitoramento e supervisão on-line [43-44].

A vacinação na gestação é um desafio de saúde pública que exige estratégias eficazes para garantir ampla cobertura e proteção para mãe e bebê. As informações claras sobre benefícios e momento adequado da vacinação por meio de materiais educativos diversos são fundamentais, assim como a comunicação direta e adaptada dos profissionais de saúde durante as consultas. Programas materno-infantis devem integrar ações e abordagens multimodais, que se mostram promissoras para reduzir a hesitação vacinal em gestantes [45-46].

Entre as limitações deste estudo, destacaram-se a ausência de avaliação sistemática da heterogeneidade dos estudos incluídos e da análise do viés de publicação, o que pode afetar a compreensão dos contextos e a representatividade das evidências. A ausência de matriz de sobreposição entre as revisões e os estudos primários constituiu outra limitação, uma vez que tal análise permitiria identificar duplicações e evitar a soma artificial de evidências. 

Como recomendações, propõe-se fortalecer a infraestrutura em áreas de baixo IDH-M, desenvolver comunicação culturalmente adaptada, implementar programas para grupos ocupacionais vulneráveis, garantir atenção à saúde materna e vacinação no pré-natal, promover educação em saúde e letramento digital, reduzir barreiras socioeconômicas e ampliar redes comunitárias e parcerias intersetoriais. Para futuras pesquisas, recomenda-se avaliar o impacto a longo prazo.

O enfrentamento à hesitação vacinal requer abordagem estratégica e integrada, que considere suas múltiplas dimensões. Esta revisão identificou intervenções com potencial para fortalecer a confiança nas vacinas e ampliar a cobertura vacinal no Brasil. Apesar das limitações nas evidências e no processo de revisão, os achados ofereceram subsídios para orientar ações nos serviços de saúde, na formulação de políticas públicas e em pesquisas. As recomendações incluíram investimentos em infraestrutura, qualificação profissional, inovação em comunicação e articulação intersetorial, com adaptações às realidades locais e foco em grupos prioritários e vulneráveis. 

## MATERIAL SUPLEMENTAR

MATERIAL SUPLEMENTAR

## Data Availability

O acesso ao banco de dados e a outros materiais utilizados na pesquisa poderão ser solicitados pelo e-mail sandra.leone@fiocruz.br.
