# Supplementary material for: Estratégias para enfrentar a hesitação em vacinação no Brasil: síntese de evidências
Source: Epidemiol Serv Saude. 2026 Oct 12;35:e20250466. [Article in Portuguese] doi: 10.1590/S2237-96222026v35e20250466.pt (PMC13647263; doi:10.1590/S2237-96222026v35e20250466.pt)
Supplement: Supplementary file 1 [file 2237-9622-ress-35-e20250466-md1-en.pdf]

Supplementary Table 1. Search strategy used in each information source (n=1,376)

**Database**	**Strategy**	**Results**
Medline (PubMed)	("Vaccine Hesitancy"[MeSH] OR "vaccine hesitancy" OR "vaccination hesitancy" OR "hesitação vacinal") OR ("Vaccine Refusal"[MeSH] OR "vaccine refusal" OR "recusa vacinal") OR ("Vaccine Acceptance"[MeSH] OR "vaccine acceptance" OR "aceitação vacinal") AND systematic[sb]	359
Embase via Elsevier	('vaccine hesitancy'/exp OR 'vaccine hesitancy' OR 'vaccination hesitancy' OR 'hesitação vacinal') OR ('vaccine refusal'/exp OR 'vaccine refusal' OR 'recusa vacinal') OR ('vaccine acceptance'/exp OR 'vaccine acceptance' OR 'aceitação vacinal') AND 'systematic review'/exp	493
Mednar	vaccine and hesitancy and review	8
Cochrane Library	(vaccine hesitancy OR vaccination hesitancy OR "hesitação vacinal") OR (vaccine refusal OR "recusa vacinal") OR (vaccine acceptance OR "aceitação vacinal") IN Title Abstract Keyword AND systematic review IN Publication Type	21
Virtual Health Library	((tw:"hesitação vacinal" OR tw:"vaccine hesitancy" OR mh:"hesitação vacinal") OR (tw:"recusa vacinal" OR tw:"vaccine refusal" OR mh:"recusa vacinal") OR (tw:"aceitação vacinal" OR tw:"vaccine acceptance" OR mh:"aceitação vacinal")) AND (mh:"revisão sistemática" OR tw:"revisão sistemática" OR tw:"systematic review")	14
Epistemonikos	(title:("vaccine hesitancy" AND "systematic review") OR abstract:("vaccine hesitancy" AND "systematic review"))	279
Health Systems Evidence	vaccine AND hesitancy AND review	17
CINAHL (EBSCO)	(TI(vaccine hesitancy OR vaccination hesitancy OR "hesitação vacinal") OR TI(vaccine refusal OR "recusa vacinal") OR TI(vaccine acceptance OR "aceitação vacinal")) AND AB(systematic review OR "revisão sistemática")	179
Article references		6
**Total**		**1,376**

Supplementary Table 2. Reasons for exclusion of full-text articles assessed for eligibility (n=7)

**Theoretical study, without results of actions to confront vaccine hesitation**
1. Bianchi FP, Stefanizzi P, Di Gioia MC, Brescia N, Lattanzio S, Tafuri S. Hesitação à vacinação covid-19 em mulheres grávidas e lactantes e estratégias para aumentar a adesão à vacinação: uma revisão sistemática e meta-análise. Expert Rev Vaccines. 2022;21(10):1443-54.<br>2. Bianchi FP, Stefanizzi P, Martinelli A, Brescia N, Tafuri S. COVID-19 vaccination hesitancy in people affected by diabetes and strategies to increase vaccine compliance: a systematic narrative review and meta-analysis. Vaccine. 2023;41(7):1303-9.
**No effectiveness data on interventions to address vaccine hesitancy**
3. Goiás. Secretaria de Estado da Saúde; Gerência de Informações Estratégicas em Saúde; Conecta-SUS. Estratégias para aumentar a cobertura vacinal: overview de revisões sistemáticas [Internet]. Goiânia: SES-GO; 2022. 9 p. Available from: https://pesquisa.bvsalud.org/portal/resource/esChatterji/biblio-1391037.<br>4. Nichol B, McCready JL, Steen M, Unsworth J, Simonetti V, Tomietto M. Barriers and facilitators of vaccine hesitancy for COVID-19, influenza, and pertussis during pregnancy and in mothers of infants under two years: an umbrella review. PLoS ONE. 2023;18(3):e0282525.<br>5. Santos AJV, Nunes ML, Moreira VG, Nascimento ACS. Fatores associados a hesitação vacinal contra covid-19: uma revisão integrativa. Rev Foco. 2023;16(5):e1992.
**Scoping review**
6. Heneghan CJ, Plüddemann A, Spencer EA, Brassey J, Rosca EC, et al. Intervenções projetadas para melhorar a adesão à vacinação: revisão de escopo de revisões sistemáticas e meta-análises (versão 1). medRxiv. 2022. Available from: https://www.medrxiv.org/content/10.1101/2022.01.26.22269890v1.full-text.
**Study involving participants under 18 years of age**
7. Suhail MK, Moinuddin A. Evaluation of strategies against vaccine hesitancy in the COVID-19 and Indian contexto: a systematic review. J Educ Health Promot. 2023;12:142. Available from: https://journals.lww.com/jehp/fulltext/2023/04280/evaluation_of_strategies_against_vaccine_hesitancy.142.aspx.
