# Supplementary material for: Estratégias para enfrentar a hesitação em vacinação no Brasil: síntese de evidências
Source: Epidemiol Serv Saude. 2026 Oct 12;35:e20250466. [Article in Portuguese] doi: 10.1590/S2237-96222026v35e20250466.pt (PMC13647263; doi:10.1590/S2237-96222026v35e20250466.pt)
Supplement: Translation

# Translation: Strategies to address vaccine hesitancy in Brazil: evidence synthesis

**DOI:** 10.1590/S2237-96222026v35e20250466.en

**Authors:** Ana Paula da Costa Marques, Vanessa Terezinha Gubert, Adriano da Costa Dias, Antonio Luiz Dal Bello Gasparoto, Liara Ferreira dos Santos, Emmanuela Maria de Freitas Lopes, Karina Morales Lopes, Thayssa Maluff de Mello, Ana Cristina Atz dos Santos, Jadher Percio, Paulo Henrique Santos Andrade, Petra Santos Castro Rangel, Roberta Silva, Aurelina Aguiar Lima, Fernando Antônio Ribeiro Gusmão-Filho, Tereza Setsuko Toma, Antonio Jose Grande, Sandra Maria do Valle Leone de Oliveira

**Affiliations:** 1 Universidade Federal de Mato Grosso do Sul, Instituto de Biociências, Campo Grande, MS, Brazil; 2 Escola Nacional de Administração Pública, Diretoria de Altos Estudos, Brasília, DF, Brazil; 3 Universidade Federal de Mato Grosso do Sul, Campo Grande, MS, Brazil; 4 Fundação Oswaldo Cruz, Campo Grande, MS, Brazil; 5 Ministério da Saúde, Brasília, DF, Brazil; 6 Universidade de Brasília, Faculdade de Ciências da Saúde, Brasília, DF, Brazil; 7 Universidade de Pernambuco, Faculdade de Ciências Médicas, Recife, PE, Brazil; 8 Instituto de Saúde, Núcleo de Evidências, São Paulo, SP, Brazil; 9 Universidade Estadual de Mato Grosso do Sul, Campo Grande, MS, Brazil

**Keywords:** Vaccine Hesitancy, Immunization, Health Strategies, Evidence-Informed Policy, Consumer Health Information

## Abstract

**Objective:**

To identify strategies to address vaccine hesitancy.

**Methods:**

A search was conducted in Medline, Embase, MedNar, Cochrane Library, Virtual Health Library (VHL), Epistemonikos, Health Systems Evidence, and CINAHL (EBSCO) between June 2024 and February 2025. The selected studies were imported into the Zotero application and subsequently into the Eppi-Reviewer application. Systematic reviews were included, with no restrictions on publication period or language, that address strategies to reduce vaccine hesitancy in an urban Brazilian setting and include adults aged 18 years and older and pregnant women. The synthesis of the results was conducted narratively. Methodological quality was assessed using the A MeaSurement Tool to Assess systematic Reviews 2 (AMSTAR 2) and the PROGnosis RESearch Strategy (PROGRESS) framework to analyze equity in vaccination strategies.

**Results:**

A total of 1,376 articles were identified, of which 48 systematic reviews were included. The evidence indicated that the strategy to address vaccine hesitancy comprises five actions: strengthening communication for different population groups; implementing continuing education programs for healthcare professionals; expanding population access to vaccines; improving responses to misinformation about vaccines; and integrating vaccination strategies for pregnant women.

**Conclusion:**

Effective addressing of vaccine hesitancy requires combined strategies and highlights the need for investments in infrastructure to expand access to vaccines, continuing education processes for healthcare professionals, communication with the population, and innovation in communication approaches and intersectoral coordination.

## Introduction

Vaccination in Brazil has shown significant progress, with emphasis on the creation of the National Immunization Program in 1973, the creation of the Brazilian Unified Health System (*Sistema Único de Saúde*, SUS) in 1988, and the integration of the National Immunization Program into the Health Surveillance Secretariat in 2003, which strengthened its structure and management capacity. Despite these continuous efforts, vaccine hesitancy has emerged as an obstacle. The COVID-19 pandemic exacerbated this problem and highlighted distrust in health authorities, socioeconomic disparities, and cultural, political, and religious factors as barriers to vaccine acceptance [1].

Vaccine hesitancy is a complex phenomenon characterized by reluctance or refusal to vaccinate despite the availability of vaccination services [2]. In addition to the factors already mentioned, the spread of misinformation, especially through social media, intensified during the pandemic [3].

In this context, public health managers must adopt flexible and context-specific approaches to ensure vaccination coverage and effectiveness [4]. Evidence syntheses support this process by assisting decision-makers in identifying alternatives aligned with institutional opportunities and adaptable to local specificities [5]. This is a dynamic process that integrates technical knowledge and the capacity to respond to the social and structural demands of the health system [6]. 

Deliberative dialogues are conducted within the process of translating evidence into public action. These dialogues, also referred to as policy dialogues, are evidence-informed processes in which stakeholders validate options to address real public health problems, considering acceptability, available resources, and the implementation context [7]. 

This evidence synthesis was developed in response to a public call for proposals in Health Evidence, funded by the Department of Science and Technology of the Brazilian Ministry of Health and the National Council for Scientific and Technological Development (*Conselho Nacional de Desenvolvimento Científico e Tecnológico*, CNPq). The objective was to identify actions/options to address vaccine hesitancy and provide input for the development of public policies. 

## Methods

### Type of study and research question

This evidence synthesis followed the “Methodological guideline: evidence synthesis for policies” of the Ministry of Health and is reported in accordance with the Reporting Checklist for Evidence Syntheses for Policies [8]. The research question was: What strategies are effective for addressing vaccine hesitancy among adults in urban settings in Brazil?

Given that systematic reviews remain the gold standard, the methodology was adapted to synthesize evidence using the PCC framework, as the study aimed to identify options rather than compare interventions. 

The search strategy was structured as follows.

- P (population, condition): adults aged 18 to 60 years, older adults aged 60 years and older, and pregnant women, including those under 18 years of age.
- C (concept): strategies implemented to promote vaccine acceptance, adherence to the immunization schedule, and vaccination campaigns.
- C (context): the Brazilian urban setting, including social, cultural, economic, and political factors.

### Eligibility criteria

All systematic reviews were included, with no restrictions on publication date or language, that address strategies to tackle vaccine hesitancy in urban settings and include adults aged 18 years and older and pregnant women. Systematic reviews from different countries were eligible, with priority given to their applicability to the Brazilian context.

Theoretical studies, narrative reviews, dissertations, theses, and opinion-based or text-based articles, as well as studies focused on rural Brazilian contexts, international vaccine hesitancy, or individuals under 18 years of age, were excluded.

Primary studies were used only to complement the analysis of equity gaps in interventions addressing vaccine hesitancy and did not constitute the main body of the synthesis, which was based on systematic reviews.

### Information sources

The searches were conducted between June 2024 and February 2025. The information sources consulted were Medline via PubMed, Embase via Elsevier, MedNar, Cochrane Library, the Virtual Health Library (VHL), Epistemonikos, Health Systems Evidence, and the Cumulative Index to Nursing and Allied Health Literature - CINAHL (EBSCO). 

### Search strategy

A pilot search was conducted in PubMed and Embase, and the search strategy was developed by AJG and SMVLO following the Peer Review of Electronic Search Strategies (PRESS) guidelines. The search strategy was built using a combination of the terms “Vaccine Hesitancy”, “Vaccine Refusal”, and “Vaccine Acceptance”, indexed or not in Medical Subject Headings (MeSH). 

The following descriptors were used: “vaccine hesitancy,” “vaccination hesitancy,” “*hesitação vacinal,*” “vaccine refusal,” “*recusa vacinal,*” “vaccine acceptance,” and “*aceitação vacinal* ”. No filters or limits were applied. The search strategy is presented in Supplementary Table 1.

### Study selection and data collection process

The articles identified through the search strategy were imported into the Zotero application and subsequently into EPPI-Reviewer. Within the software, duplicate citations were removed, and the entire screening and study selection process was conducted independently by four reviewers. The list of excluded studies and the reasons for exclusion are presented in Supplementary Table 2.

All stages of title and abstract screening, full-text review, and data extraction were performed by two pairs of researchers (APCM and EMFL; ACD and KML). Disagreements were resolved by a third researcher (AJG).

Data extraction was performed manually using a spreadsheet developed by the research team and validated by the stakeholders who commissioned the study, based on Appendix E - Synthesis of Studies by Option from the “Methodological Guideline: Evidence Synthesis for Policies” [8]. The study spreadsheet included the following fields: author/year; option; study objective; study characteristics; main findings-review context, primary and secondary outcomes, conclusion, potential benefits and harms; study period; and methodological quality assessment. 

Based on data extracted into an Excel spreadsheet, options for addressing vaccine hesitancy among adults living in urban areas of Brazil were identified and subsequently validated by stakeholders through a policy dialogue. 

### Assessment of risk of bias and equity in vaccination strategies

Methodological quality and risk of bias were assessed by AJG and ACD using the A MeaSurement Tool to Assess Systematic Reviews 2 (AMSTAR 2) [9], which comprises 16 items. Each item was rated as “yes,” “no,” or “partial yes,” according to the degree of compliance with the methodological criteria. A “yes” response indicated full compliance with the item; “no” indicated that assessment was not possible due to insufficient information; and “partial yes” indicated partial compliance with the criterion. The scores were subsequently grouped and categorized into levels of methodological confidence: high (0-1 non-critical item), moderate (>1 non-critical item), low (1 critical item), or critically low (>1 critical item).

Primary studies were included only to complement the equity analysis of strategies addressing vaccine hesitancy and were not incorporated into the main synthesis of systematic reviews. The PROGnosis RESearch Strategy (PROGRESS) framework [10] was used to help identify barriers and opportunities to promote equity in vaccine access. The following dimensions were assessed: place of residence, race, occupation, gender/sex, education, socioeconomic status, and social capital. This analysis was conducted independently by AJG and ACD.

### Synthesis method

Data were summarized through a narrative synthesis, with the identified strategies grouped into predefined thematic categories:

- option 1 - strengthen communication for different population groups;
- option 2 - implement continuing education programs for health professionals;
- option 3 - expand population access to vaccines;
- option 4 - improve responses to vaccine misinformation; and
- option 5 - integrate vaccination strategies for pregnant women.

This approach enabled the findings to be organized descriptively, highlighting the types of interventions, target populations, and implementation contexts.

The synthesis considered both the frequency with which each strategy was reported across the included studies and the observed effects on vaccine uptake. The evidence was presented qualitatively, without meta-analysis, due to the heterogeneity of methodological designs, study populations, and outcome measures. In addition, contextual factors influencing the effectiveness of the strategies, including cultural, social, and economic aspects, were highlighted.

## Results

The initial search identified 1,376 records, of which 1,055 reviews were screened after duplicates were removed. During the full-text assessment of 55 articles, 7 were excluded and are listed in Supplementary Table 2. A total of 48 systematic reviews were included ( Figure 1). 

Published between 2012 and 2024, the reviews were conducted in high-income countries and also drew on evidence from middle- and low-income countries, such as Brazil. The vaccines addressed included COVID-19, influenza, human papillomavirus (HPV), hepatitis B, pertussis, and pneumococcal vaccines, with a focus on diverse populations and settings (Table 1). 

Each review described one to four strategies to address vaccine hesitancy, with combinations of strategies being the most effective. The five main strategies identified were: strengthening communication with different population groups; implementing continuing education programs for health professionals; expanding access to vaccines; addressing vaccine misinformation; and integrating vaccination strategies for pregnant women. These strategies were validated with the stakeholders who commissioned the study through a policy dialogue conducted at the conclusion of the evidence synthesis developed for the National Immunization Program.

A wide range of strategies has been adopted to strengthen communication, promote vaccine uptake, and mitigate vaccine hesitancy [42]. The use of behavior change theories, particularly the Health Belief Model, has been prominent. Across social media platforms, educational posts predominate, followed by discussion groups, interactive websites, and personalized reminders [39]. Honest and transparent communication, combined with information about vaccine benefits and potential adverse events, enhances the credibility of vaccination campaigns [34]. Direct engagement may be effective among populations resistant to vaccination [19]. 

**Figure 1:** Study selection and inclusion process

Communication that is sensitive to cultural and personal nuances is more effective than strategies based exclusively on epidemiological and medical knowledge [42]. The involvement of community and religious leaders strengthens trust and vaccine uptake, particularly among ethnic minority and migrant populations. Interventions incorporating behavior change techniques, combined with tailored messages and support from community organizations, have proven effective in reaching these groups [34]. 

Multifaceted strategies, such as posters, leaflets, internal communication, and active vaccine offering at the locations where these materials are distributed, significantly increased influenza vaccination rates. Videos, virtual reality, and mobile applications make communication more dynamic and engaging, particularly among younger populations, and complement traditional approaches, enhancing engagement and vaccine uptake [19]. Notable interventions include educational activities for service users, vaccination record tracking by community health workers, and simultaneous interventions targeting health facilities, health professionals (parent education, reminders, audit and feedback), and family environments (telephone calls) [59].

Among social media interventions, educational posts, dialog-based interactive groups, interactive websites, and personalized reminders are the most commonly used. These strategies were informed by nudge theory [57]. This theory proposes subtly influencing choices, without restricting options or imposing obligations, by introducing small changes to the environment or context in which decisions are made. 

**Table 1:** Characteristics of included studies (n=48)

Reference	Option	Main findings	Methodological quality
[11]	3 and 4	It is recommended to build trust, reduce physical barriers, and improve communication and transparency regarding vaccine development, with the support of healthcare professionals, religious leaders, and community leaders, in order to increase access and uptake among ethnic minorities and migrant populations.	High
[12]	1	To increase vaccine acceptance, sociocultural, historical, and political contexts should be considered, prioritizing communication, community engagement, and culturally inclusive resources, with tailored strategies that reduce hesitancy and increase vaccination uptake.	Moderate
[13]	1	Digital technology-based strategies have a modest but positive impact on vaccine acceptance and completion of vaccination schedules compared with non-digital interventions.	High
[14]	1	Access to electronic health records moderately increases vaccination uptake, and its combination with digital communication tools enhances this effect, outperforming electronic health record access alone.	Moderate
[15]	3	Personalized communications and message reminders increase vaccination adherence, while communicating uncertainties about the vaccine does not reduce the intention to get vaccinated, but can have a negative impact.	High
[16]	2 and 4	Future policies should address population concerns, promote understanding of vaccines, counter misinformation, and increase acceptance, with evidence-based training on vaccines and vaccination strategies for healthcare professionals.	High
[17]	5	Several strategies were found to be effective, including vaccination reminders in prenatal health records, midwife-led vaccination, and education and information provision for healthcare teams and service users.	Moderate
[18]	5	Educational interventions, such as leaflets, websites, and brief individual sessions, increase influenza vaccination coverage among pregnant women, with higher acceptance of the influenza vaccine.	Moderate
[19]	1 and 2	Promotional communication interventions are more effective when they are personalized, use multiple channels, involve healthcare professionals, are interactive, and address public concerns.	Moderate
[20]	1	Barriers such as language, poor access to healthcare, and mistrust in the healthcare system can be mitigated through greater resource allocation and engagement. Promoting policies that ensure federal funding for health services and the adoption of Advisory Committee on Immunization Practices guidelines would improve vaccination rates among refugees, immigrants, and migrants.	Moderate
[21]	3	Among healthcare professionals, lectures, informational materials, mobile units, extended hours, and leadership engagement are effective strategies. Combining these strategies with motivational incentives strengthens adherence, particularly in contexts where mandatory vaccination is controversial.	Moderate
[22]	1 and 2	Effective interventions to increase vaccine uptake require personalized and culturally sensitive approaches. The ineffectiveness of one-size-fits-all models highlights the need for tailored actions that consider cultural norms, beliefs, and ideologies.	High
[23]	1	The reviewed studies included interventions with diverse content and approaches implemented across different settings and targeting various populations. Few interventions were directly aimed at individuals experiencing vaccine hesitancy.	Moderate
[24]	1, 2 and 5	Multicomponent interventions adapted to the needs of specific ethnic minority groups are effective in increasing vaccine acceptance, particularly when they emphasize awareness-raising and engagement with community organizations.	High
[25]	2	Multidimensional community-, family-, and healthcare-based interventions, combined with educational strategies, effectively improve vaccination coverage. Systematic implementation may be relevant for reaching under-vaccinated communities.	High
[26]	1	Emails increase vaccine acceptance compared with no intervention. Combined and personalized reminder methods are more effective. Personalization and integration of different strategies are crucial to optimize outcomes.	High
[27]	2 and 3	Peer-education initiatives have the potential to improve vaccine acceptance, particularly when they combine peers and health professionals, and when implemented through targeted, long-term actions.	High
[28]	2	Strategies varied widely in delivery mode and behavioral change techniques. After interventions, influenza, pneumococcal, and herpes zoster vaccination rates increased by 16.6% over a 12-16 month period.	Moderate
[29]	2	Herd immunity represents a significant benefit of vaccination at the population level. Communicating this benefit effectively is crucial, as evidence suggests that clear messaging about herd immunity may increase vaccination intentions.	High
[30]	4	Social media has the potential to disseminate information and promote vaccination; however, its effectiveness requires active and responsible monitoring by authorities and must be combined with other strategies due to the complexity of vaccine acceptance.	Moderate
[31]	3	Behavioral interventions, both financial and non-financial, have a modest positive effect on vaccine uptake, with similar impacts between single and combined approaches. Timing of implementation does not significantly affect outcomes, except for community-based interventions, which may lose effectiveness as the COVID-19 pandemic evolves.	High
[32]	2	When pharmacists are involved in the immunization process, regardless of their specific role or vaccine type, vaccination coverage is higher.	High
[33]	3	Multicomponent and dialog-based interventions are more effective; however, given the complexity of vaccine hesitancy and limited evidence, strategies should be adapted to the target population, their reasons for hesitancy, and the specific context.	High
[34]	1, 2, 3 and 5	The most effective strategies include clear information, personalized reminders, improved access to vaccination, and encouragement from healthcare professionals. In the United Kingdom, focusing on information relevance and improving access has proven particularly effective.	High
[35]	3	Barriers to vaccination vary by infectious disease and population; the main motivators are self-protection and protection of family and community. For influenza, more effective strategies focus on convenience and trust, addressing issues such as cost and education.	Moderate
[36]	2, 3 and 5	Vaccine acceptance among pregnant women is facilitated by healthcare professional recommendations, knowledge, vaccine confidence, multiple antenatal visits, and strategies such as financing, reminders, service integration, community engagement, training, and education.	Moderate
[37]	5	Postpartum vaccination uptake is strengthened by healthcare provider recommendations, tailored interventions, vaccination in maternity or community settings, and education focused on positive reinforcement.	Moderate
[38]	1, 2 and 5	Providing information on vaccine risks and safety, debunking misconceptions, using reminders and mixed campaigns, and delivering short, clear, audience-tailored messages increases acceptance and strengthens positive vaccination beliefs.	Low
[39]	1 and 2	Educational messages increase knowledge; however, dialog-based social media interventions grounded in behavior change theory and adapted to the target audience show greater potential to promote vaccination and reduce hesitancy.	Moderate
[40]	4	Social media interventions are an emerging and promising field to increase vaccine acceptance, where message framing, source credibility, and social network structure are key determinants, making optimization essential.	Moderate
[41]	2	Applications, games, and simulations are promising interventions to improve knowledge and confidence among healthcare professionals and students regarding vaccination, with better results when combined with user-targeted actions.	High
[42]	1,2 and 3	Diverse and combined communication strategies, including mass media and personalized approaches with improved service delivery, show potential to increase vaccine acceptance, particularly for influenza among older adults.	Moderate
[43]	1 and 3	Participants were more likely to be vaccinated when incentivized with monetary rewards. Other lottery-based incentives and persuasive messages had little or no significant effect.	Moderate
[44]	1 and 2	Effective vaccination promotion strategies include communication (webinars, social media, and educational materials), partnerships among state authorities, universities, and community leaders, as well as delivery interventions such as virtual municipal services, confidential hotlines, educational materials, and vaccination offers during routine medical visits.	Moderate
[45]	1 and 2	Strengthening the role of healthcare professionals in promoting vaccination is crucial. Strategies such as standing orders, reminders, and recall systems are effective. Community pharmacies, especially in remote areas, have the potential to improve vaccination coverage.	Moderate
[46]	1 and 2	Game-based interventions generally increase knowledge about vaccination; however, their impact on vaccination uptake is limited. However, the impact on vaccination adherence itself is limited. Avatar customization and collaboration in games have been shown to effectively engage participants. and in-Game collaboration have proven effective at engaging players.	Moderate
[47]	1	Videos, virtual reality, and applications with social networking or gamification elements are more effective than printed materials in increasing knowledge, confidence, and engagement in vaccination.	High
[48]	1	Vaccine *chatbots* have been used to provide factual information, schedule vaccinations, send reminders, and address misinformation, *showing* potential to positively influence vaccine-related attitudes.	Moderate
[49]	1	Multicomponent strategies are more effective, particularly when targeting multiple determinants of vaccination behavior.	Low
[50]	5	Clear and personalized recommendations for users, education on vaccine safety, encouragement of vaccine provision by providers, financial incentives, electronic health records, and vaccine availability at the policy level are effective.	High
[51]	1	Shared decision-making interventions significantly increased vaccine acceptance compared with control groups.	Moderate
[52]	1, 2 and 3	Community-based interventions, financial incentives, and technology-based health literacy programs increased immunization service uptake. Media-based interventions were not effective in reducing vaccine hesitancy.	High
[53]	1 and 2	Non-financial interventions, such as appointment reminders and *opt-out* scheduling systems, appear to encourage vaccine acceptance. Other approaches, such as multimodal interventions and infographics, also show potential to address vaccine hesitancy.	High
[54]	3	Key strategies to promote vaccination include understanding and collaborating with communities, tailoring messages, addressing systemic trauma and social health gaps, and conducting advance logistical planning.	High
[55]	2	Multicomponent interventions were effective in increasing vaccination rates, followed by personalized reminders, although less effective than standing orders or patient reminders.	High
[56]	1	Effective strategies against vaccine hesitancy include normative messaging, identity-congruent sources, interactive channels (such as *chatbots* and videos), information on safety and efficacy, collective appeals with social pressure, and support from political leaders.	High
[57]	1	Nudge-based interventions increased COVID-19 vaccine uptake, particularly when combining multiple components (e.g., social norms and reminders), although effects diminished over time.	High
[58]	4	Vaccine-related misinformation was addressed in five stages: identification of misinformation, regulation of content producers and distributors, disruption of production and dissemination, support for target audiences, and dissemination of reliable information.	High

The success of communication strategies depends on integrating multiple approaches that account for cultural, technological, and social factors. Combining traditional methods with digital technologies, together with the cultural and linguistic adaptation of messages, facilitates outreach to different population groups and promotes meaningful changes in health-related behaviors.

Continuing health education is a policy of the Brazilian Unified Health System (SUS) that should be used as a strategy to address vaccine hesitancy. Health professional training should develop communication skills that enable professionals to convey information clearly and objectively to the public and to engage other professionals in prescribing and recommending vaccines included in the National Immunization Program [59]. Personal narratives combined with clear and accessible medical information have been effective in engaging diverse audiences, ranging from university students to the general population [39]. 

Personalized reminders for physicians, as a form of passive education, have demonstrated positive effects [55]. Brief messages that focus on risk reduction, reinforce the social benefits of vaccination, and address the beliefs and concerns of target populations, such as vaccine safety, increase behavioral impact [38].

Difficulty accessing health services contributes to vaccine hesitancy. Improving signage for vaccination rooms and publicizing vaccination clinic opening hours are relevant measures [15]. Mobile units, vaccination clinics, and extended service hours improve access for workers and remote communities [21,34]. Assessment of barriers and facilitators indicates that vaccination delivered in non-traditional settings, such as schools, workplaces, and community centers, has proven effective [35].

Misinformation is one of the greatest obstacles to vaccination campaigns, making it essential to address it to reduce vaccine hesitancy [25]. The active and trustworthy engagement of official institutions on social media, through transparent responses, the promotion of accurate information, and fact-checking by health professionals, is fundamental to countering misinformation and increasing confidence in vaccines [30,40]. To support these strategies, it is necessary to establish surveillance systems, regulate information production, and develop measures to prevent the circulation of false content [58].

Vaccination of pregnant and breastfeeding women remains a public health challenge, with a coverage rate of 48.4%, attributed to lack of information, perceptions of lack of vaccine safety, and fear of adverse events affecting both mother and child. In the case of COVID-19, in addition to traditional concerns, pregnant women reported distrust of vaccine efficacy and safety, influenced by the rapid pace of vaccine development and the perceived influence of the pharmaceutical industry on public policy [60]. 

Strategies that combine affective messages, such as videos and books, with cognitive approaches increase knowledge and vaccination intention, with video-based interventions being the most effective [24]. Multimodal educational strategies, including oral, written, and visual information, such as posters, may increase vaccination rates. 

The training of health professionals is essential for addressing vaccine hesitancy. Professional education should include strategies to address pregnant women’s concerns and strengthen confidence in vaccination, given that healthcare provider recommendations are a key determinant of vaccine uptake [37]. All communication should be clear, emphasize benefits for the infant, and be tailored to the stage of pregnancy, taking into account women’s needs and concerns [34]. 

Facilitating access to vaccination, particularly in settings frequented by pregnant women, such as maternity hospitals, promotes vaccine uptake [37]. Educational interventions, text messaging, and combined approaches have demonstrated positive effects on maternal immunization in low-income settings [18,36]. 

The assessment of the 48 systematic reviews using the AMSTAR 2 tool indicated that 50.0% were of high methodological quality, 45.8% of moderate quality, and 4.2% of low quality. This distribution strengthens the robustness of the synthesis, although methodological limitations should be considered when interpreting the findings. Table 2 presents the risk-of-bias assessment. 

**Table 2:** Assessment of risk of bias and methodological quality (n=48)

	Sixteen questions that make up the AMSTAR 2 Tool																
Author, year	1	2	3	4	5	6	7	8	9	10	11	12	13	14	15	16	Classification
Abba-Aji, 2022 [11]																	High
Adeagbo, 2022 [12]																	Moderate
Atkinson, 2019 [13]																	High
Balzarini, 2020 [14]																	Moderate
Batteux, 2022 [15]																	High
Begum, 2024 [16]																	High
Bisset and Paterson, 2018 [17]																	Moderate
Brillo, Tosto and Buonomo, 2023 [18]																	Moderate
Cairns, 2012 [19]																	Moderate
Daniels, 2022 [20]																	Moderate
De Koning, 2024 [21]																	Moderate
Desjardins, 2023 [22]																	High
Dubé, Gagnon and MacDonald, 2015 [23]																	Moderate
Ekezie, 2023 [24]																	High
Fallucca, 2024 [25]																	High
Frascella, 2020 [26]																	High
Gobbo, 2023 [27]																	High
Gosselin Boucher, 2019 [28]																	Moderate
Hakim, 2019 [29]																	High
Hansen, Baiju and Gabarron, 2023 [30]																	Moderate
Huang, Huang and Yu, 2023 [31]																	High
Isenor, 2016 [32]																	High
Jarrett, 2015 [33]																	High
Kafadar, 2024 [34]																	High
Kalunga, 2023 [35]																	Moderate
Khan, 2024 [36]																	Moderate
Konstantinou, 2024 [37]																	Moderate
Lawes-Wickwar, 2021 [38]																	Low
Li, Wood and Kostkova, 2022 [39]																	Moderate
Limaye, 2021 [40]																	Moderate
Lo Moro, 2023 [41]																	High
MacDonald, 2013 [42]																	Moderate
Mardi, 2022 [43]																	Moderate
Masterson, Anderson and Savoia, 2024 [44]																	Moderate
Mohammed*,* 2021 [45]																	Moderate
Ou, 2024 [46]																	Moderate
Panickar, 2023 [47]																	High
Passanante, 2023 [48]																	Moderate
Perroud, 2022 [49]																	Low
Razai, 2023 [50]																	High
Scalia, Durand and Elwyn, 2022 [51]																	Moderate
Singh, 2022 [52]																	High
Terrell, Alami, and Krewski, 2023 [53]																	High
Tinessia, 2024 [54]																	High
Wennekes, 2024 [55]																	High
Xia and Nan, 2024 [56]																	High
Zhang and Jin, 2024 [57]																	High
Zhao*,* 2023 [58]																	High

-Yes

-Partial yes

-No

-Not applicable

The evaluation of vaccination policies and programs must consider their effects on social inequalities and ensure access to reliable and understandable information regardless of origin or social circumstances. Overcoming historical, cultural, and technological barriers is essential to achieving equity, and the PROGRESS framework helps identify barriers and opportunities [60]. 

Regions with low Municipal Human Development Index (MHDI) scores face challenges with infrastructure and connectivity at vaccination facilities, whereas areas with higher MHDI scores generally have better logistical conditions. Maranhão, despite having one of the lowest Human Development Index among Brazilian states, achieved improvements in vaccination coverage during the COVID-19 pandemic, driven by broader Family Health Strategy (*Estratégia Saúde da Família*, ESF) coverage and the decentralization of health services [61]. Immunization programs in remote communities have proven essential for promoting equity [62].

Minority populations and ethnic groups face longstanding cultural and historical challenges related to distrust of clinical interventions resulting from exclusion and discrimination. Communication should be adapted to cultural and linguistic contexts to ensure effective understanding [16]. Strategies involving community participation and the use of clear, evidence-based educational materials strengthen trust in and acceptance of vaccination among pregnant women [10]. Actions such as active outreach and point-of-care vaccination have been effective among vulnerable populations [11].

Pregnant women have specific vulnerabilities that require targeted strategies due to the immunological changes associated with pregnancy. The quality of prenatal care directly influences vaccination rates, with more pronounced inequalities among women with lower income and educational attainment. Vaccine hesitancy in this group stems from a lack of reliable information, beliefs regarding the lack of vaccine safety, and fear of adverse effects [30]. Educational materials, one-on-one sessions, and digital resources have proven effective in increasing vaccine uptake among this population and promoting equity in maternal and child healthcare [18,63]. Promoting greater equity in maternal and child healthcare requires policies that address socioeconomic and educational barriers, improve access, and strengthen health systems to protect pregnant women and newborns [10,18,61,63].

Educational level is strongly associated with vaccine uptake, highlighting the need for accessible materials and simplified language for populations with low literacy levels [64]. Digital health literacy has emerged as a strategic tool, particularly during pandemics [65]. Conversation circles have proven effective in promoting vaccination among vulnerable groups, such as patients and accompanying persons in oncology service waiting rooms [1]. 

Promoting social participation is essential for addressing inequalities in access to healthcare and supporting vulnerable groups [53]. From a PROGRESS perspective, recommendations include strengthening infrastructure in areas with low MHDI scores; developing culturally sensitive and tailored communication strategies; implementing programs for vulnerable occupational groups; ensuring maternal healthcare and vaccination during prenatal care; promoting health education and digital health literacy; reducing socioeconomic barriers to access; and expanding community networks and intersectoral partnerships. Equitable access to vaccination requires consideration of the multiple barriers faced by different population groups.

## Discussion

Cultural, social, economic, and informational factors influence vaccine hesitancy. To address this scenario, it is suggested to strengthen communication with diverse population groups, implement continuing education for health professionals, expand access to vaccines, address vaccine misinformation, and integrate specific strategies for pregnant women [40-41].

Strengthening communication requires the involvement of community leaders and adaptation of language to local values. Messages should emphasize both collective and individual benefits of vaccination, using emotional appeals and political figures to create identification and trust [42]. Media campaigns, community education, and expanded access to vaccination services are essential, as are the ongoing outreach efforts conducted by health teams. Digital technologies, such as electronic reminders, online platforms, short message service (SMS), social media, and *chatbots*, facilitate vaccine appointment scheduling and provide clear information on vaccine safety, reaching broad and diverse audiences.

Continuing education programs for health professionals help address vaccine hesitancy by using clear visual materials and reliable information sources, thereby strengthening trust between professionals and users. These programs should seek to increase competence for context-adapted interventions, including the use of presumptive announcements during consultations. Continuing education programs must involve health professional leaders and prioritize in-person educational activities. Personalized reminders combined with tools that facilitate work within health services are also recommended.

To address vaccine hesitancy, it is necessary to expand access to vaccines and improve service convenience by extending vaccination hours, sending personalized messages, using mobile units, and offering vaccinations in non-traditional settings, such as schools and workplaces. Opportunistic vaccination, workplace vaccination, and monetary incentives also increase uptake.

Misinformation is one of the greatest obstacles to vaccination campaigns. Several strategies have proven effective in addressing this challenge, including community engagement, participation of religious and community leaders, automated scheduling, personalized communications, strategies designed to avoid the backfire effect, and language tailored to public concerns, together with online regulation, monitoring, and oversight [43-44].

Vaccination during pregnancy is a public health challenge that requires effective strategies to ensure broad coverage and protection for both mother and infant. Clear information about vaccine benefits and appropriate timing, delivered through diverse educational materials, is essential, as is direct, tailored communication from health professionals during consultations. Maternal and child health programs should integrate actions and multimodal approaches, which appear promising for reducing vaccine hesitancy among pregnant women [45-46].

Among the limitations of this study, the absence of a systematic assessment of heterogeneity among included studies and of publication bias analysis may have affected the understanding of contexts and the representativeness of the evidence. The absence of an overlap matrix between reviews and primary studies was another limitation, as such an analysis would allow the identification of duplicate evidence and help avoid artificial inflation of the evidence base. 

As recommendations, the study proposes strengthening infrastructure in areas with low MHDI scores, developing culturally adapted communication strategies, implementing programs for vulnerable occupational groups, ensuring maternal healthcare and vaccination during prenatal care, promoting health education and digital health literacy, reducing socioeconomic barriers, and expanding community networks and intersectoral partnerships. For future research, it is recommended to assess the long-term impact.

Addressing vaccine hesitancy requires a strategic and integrated approach that considers its multiple dimensions. This review identified interventions with the potential to strengthen confidence in vaccines and expand vaccination coverage in Brazil. Despite limitations in the evidence base and the review process, the findings provide support for actions in health services, public policy formulation, and future research. Recommendations include investments in infrastructure, workforce development, communication innovation, and intersectoral coordination, with adaptation to local realities and a focus on priority and vulnerable groups. 

## SUPPLEMENTARY MATERIAL

SUPPLEMENTARY MATERIAL

## Data Availability

The dataset and other materials used in this study are available from the corresponding author upon reasonable request at sandra.leone@fiocruz.br.
